# 

**Published:** 1894-07

**Authors:** 


					﻿NOTICE
Af^^ficlés^»rpul>lication, bPkf >^r review, /><<««'neea eommwnieatione, etc.,
snfa^b^peg^lv Editor,	Street, Philadelphia.
l^utM^oere tri Znj^\<>W^ that this Journal will be sent until arrears
are &sid and it i^^^jSfeb discontinued.
				

